# Visualization of dynamics in coupled multi-spin systems

**DOI:** 10.5194/mr-3-145-2022

**Published:** 2022-08-09

**Authors:** Jingyan Xu, Dmitry Budker, Danila A. Barskiy

**Affiliations:** 1 Institut für Physik, Johannes Gutenberg Universität Mainz, 55128 Mainz, Germany; 2 Helmholtz Institut Mainz, 55128 Mainz, Germany​​​​​​​; 3 GSI Helmholtzzentrum für Schwerionenforschung, Darmstadt, Germany; 4 Department of Physics, University of California at Berkeley, Berkeley, California 94720-7300, USA​​​​​​​

## Abstract

Since the dawn of quantum mechanics, ways to visualize spins and their interactions have attracted the attention of researchers and philosophers of science. In this work we present a generalized measurement-based 3D-visualization approach for describing dynamics in strongly coupled spin ensembles. The approach brings together angular momentum probability surfaces (AMPS), Husimi Q functions, and DROPS (discrete representations of operators for spin systems) and finds particular utility when the total angular momentum basis is used for describing Hamiltonians. We show that, depending on the choice of a generalized measurement operator, the plotted surfaces either represent probabilities of finding the maximal projection of an angular momentum along any direction in space or represent measurable coherences between the states with different total angular momenta. Such effects are difficult to grasp by looking at (time-dependent) numerical values of density-matrix elements. The approach is complete in a sense that there is one-to-one correspondence between the plotted surfaces and the density matrix. Three examples of nuclear spin dynamics in two-spin systems are visualized: (i) a zero- to ultralow-field (ZULF) nuclear magnetic resonance (NMR) experiment in the presence of a magnetic field applied perpendicularly to the sensitive axis of the detector, (ii) interplay between chemical exchange and spin dynamics during high-field signal amplification by reversible exchange (SABRE), and (iii) a high-field spin-lock-induced crossing (SLIC) sequence, with the initial state being the singlet state between two spins. The presented visualization technique facilitates intuitive understanding of spin dynamics during complex experiments as exemplified here by the considered cases. Temporal sequences (“the movies”) of such surfaces show phenomena like interconversion of spin order between the coupled spins and are particularly relevant in ZULF NMR.

## 1 Introduction

The evolution of spins in nuclear magnetic resonance (NMR) experiments can be highly complex and non-intuitive. Thus, approaches to visualize spin dynamics of nuclear multi-spin systems are sought for both communication and research purposes. Most NMR textbooks visualize the motion of a single spin-1/2 (or an ensemble of spins) using the Bloch vector eg.

In more complex cases such as coupled spin systems, visualization is significantly less straightforward, and the discussion is often assisted by drawing energy-level diagrams. These diagrams do not provide dynamic information but can be useful for representing populations of various spin states eg. Spin dynamics can be visually presented via coherence-transfer pathways, i.e., graphical conventions that show the path through different levels of coherences as a function of time during the implementation of high-field NMR pulse sequences. These pictures can be used to derive phase cycles and to obtain pure-phase NMR signals via symmetry properties of the phases appreciated from the graphical representations [Bibr bib1.bibx10].

Recently, [Bibr bib1.bibx15] introduced an approach of so-called “DROPS” (DROPS: discrete representation of operators for spin systems) for visualizing spin operators [Bibr bib1.bibx15]. This visualization approach is based on plotting 3D colored shapes (droplets) that correspond to linear combinations of spherical harmonics. The DROPS approach can represent interactions in Hamiltonians and in propagators as well as states of the density matrix [Bibr bib1.bibx15]. The DROPS approach with a LISA basis (with defined linearity, subsystem, and auxiliary criteria) reflects the rotational symmetry of individual spins and can be useful for understanding high-field NMR experiments where each spin can be addressed individually (for example, when a strong magnetic field breaks the isotropic symmetry of the system). While the DROPS approach could be generalized to isotropic systems by using multiple tensor operator basis, it is challenging to extract the information corresponding to measurable properties from the DROPS with complicated colors. To address these limitations, we introduce a generalized approach based on angular momentum probability surfaces (AMPS) [Bibr bib1.bibx4].

The AMPS were introduced to visualize the angular momentum state of atoms. It is worth pointing out that AMPS are a particular case of the s -parametrized phase-space functions with s=-1 , also known as the Husimi Q function first published in 1940; see Appendix G [Bibr bib1.bibx16].

To understand the physics behind the plotting, one can assume that the spin ensemble is measured by a Stern–Gerlach experiment, and the probability of finding the maximal projection state is plotted as a surface; the radius from the origin of the coordinate system to a point on the surface represents the measured maximal probability along that direction. The “measurements” are conducted with the measurement device rotated by Euler angles with the z – y – z convention (ϕ,θ,0) . The AMPS can be constructed by setting the radius of the surface along the direction (θ,ϕ) equal to the measured probability. Despite their utility in atomic physics, AMPS have limitations for use in NMR. For example, they do not represent coherences connecting states with different total angular momenta (including states of the same total angular momentum quantum number F but belonging to different manifolds) which play a crucial role in NMR of multi-spin systems. In this study, we demonstrate that such coherences can also be visualized assuming a generalized form of the measurement operator, with the measurement device rotated along various directions. In addition, we show that one of the selected measurements is directly related to the measured zero- to ultralow-field (ZULF) NMR signal. As a result, our approach is helpful in understanding the complicated splittings in ZULF NMR spectra. The approach presented in this work constitutes convenient means for visualizing complex dynamics in multi-spin systems exemplified here for pairs of nuclear spins-1/2.

## 2 Results

In our visualization approach, a density matrix $\hat{\rho }$ is represented by the action of a measurement operator ${\hat{O}}_{\hat{n}}$ :

1 $$r_{\hat{n}}=\mathrm{Tr}(\hat{\rho }\;{\hat{O}}_{\hat{n}}).$$

Here, $r_{\hat{n}}$ indicates the distance of the plotted surface point to the origin along $\hat{n}$ (Fig. [Fig Ch1.F1]a). In order to find the exact form of the operator ${\hat{O}}_{\hat{n}}$ , a measurement is first defined as ${\hat{O}}_{\hat{z}}$ (note that the subscript $\hat{z}$ denotes the direction of plotting the result of the measurement and does not specify the direction of the measured property). For example, ${\hat{O}}_{\hat{z}}$ could be $\left({\hat{I}}_{1x}{\hat{I}}_{2y}-{\hat{I}}_{1y}{\hat{I}}_{2x}\right)$ ; see below. The operator ${\hat{O}}_{\hat{n}}$ then represents the observable obtained via global rotation ${\hat{R}}_{\hat{z}\to \hat{n}}$ , which brings $\hat{z}$ to $\hat{n}$ :

2 $${\hat{O}}_{\hat{n}}={\hat{R}}_{\hat{z}\to \hat{n}}\;{\hat{O}}_{\hat{z}}\;{\hat{R}}_{\hat{z}\to \hat{n}}^{-1}.$$

**Figure 1 Ch1.F1:**
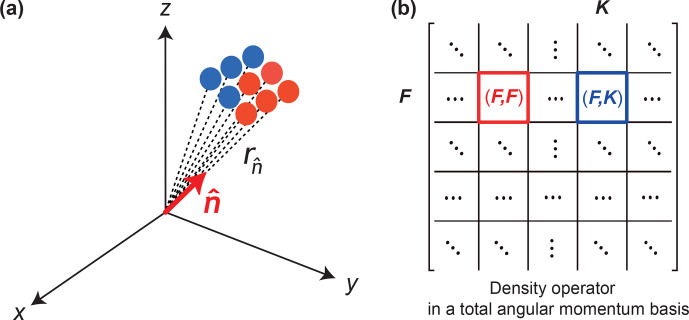
**(a)** The visualized surface is a collection of points plotted at a distance $r_{\hat{n}}$ from the origin along the direction $\hat{n}$ (red color represents a positive number and blue color represents a negative number). **(b)** Density matrix written on a total angular momentum basis. In such a basis, the density operator is decomposed into blocks according to F (total angular momentum quantum number); the diagonal block is denoted as (F,F) and the off-diagonal block is denoted as (F,K) . In the following figures we keep the same axis angles.

There is one important caveat. The observable ${\hat{O}}_{\hat{z}}$ must remain unchanged under the rotation about $\hat{z}$ ; otherwise, the way in which the rotation is performed will affect the plotted result. To avoid ambiguity, only zero-quantum operators can be used as the measurement operators since they fulfill the required property of invariance under rotation (see Appendix A). Moreover, the operator ${\hat{O}}_{\hat{n}}$ must be Hermitian for a measurement $r_{\hat{n}}$ to be real. Under such constraints, one may still obtain a negative value for $r_{\hat{n}}$ ; thus, color is introduced as an additional “degree of freedom”. As a convention, in this work we set the distance of the surface along $\hat{n}$ as the amplitude of $r_{\hat{n}}$ and set the color of the surface along $\hat{n}$ to be red if $r_{\hat{n}}$ is positive and blue if $r_{\hat{n}}$ is negative. If the observable operators are not set to be Hermitian, a color map is necessary to express ( 0 , 2π ) phase dependence of the calculated expectation value, which is, generally, a complex number [Bibr bib1.bibx15].

In the following discussion, we focus on constructing measurement operators $\left\{{\hat{O}}_{\hat{n}}\right\}$ for isotropic or nearly isotropic coupled nuclear spin systems. Nuclear spin Hamiltonians for such systems remain unchanged (or changed insignificantly) under global rotation. Therefore, we choose to work in the total angular momentum basis which is defined by the eigenstates of the operators ${\hat{F}}_{z}$ and $(\hat{F},\hat{F})={\hat{F}}_{x}^{2}+{\hat{F}}_{y}^{2}+{\hat{F}}_{z}^{2}={\hat{F}}^{2}$ :

3 $$\begin{array}{rl} & {\hat{F}}_{z}|F,m\rangle =m|F,m\rangle ,\\ & {\hat{F}}^{2}|F,m\rangle =F(F+1)|F,m\rangle , \end{array}$$

where ${\hat{F}}_{\alpha }$ is a total spin projection operator ( α=x,y,z ), ${\hat{F}}_{\alpha }=\sum_{i=1}^{N}{\hat{I}}_{i\alpha }$ for N spins-1/2, and $\hat{F}=({\hat{F}}_{x},{\hat{F}}_{y},{\hat{F}}_{z})$ is the total angular momentum vector operator.

Now we write the density operator in the total angular momentum basis which decomposes it into blocks according to the total angular momentum quantum number F (Fig. [Fig Ch1.F1]b). Since blocks of the density matrix transform differently under global rotations, visualizations (Eq. [Disp-formula Ch1.E1]) of different blocks are introduced separately to capture full information about the system. We denote diagonal blocks as (F,F) and non-diagonal blocks as ( F,K ). For the system consisting of N nuclear spins, there are in total $h=C_{N}^{N/2}$ (binomial coefficient) diagonal blocks for the even N and $h=C_{N}^{(N-1)/2}$ diagonal blocks for the odd N . In order to reproduce the full spin dynamics of a general density matrix using our method, a total number of $h^{2}$ visualized surfaces is required. In practice, depending on the specific initial state and interactions in the system, a lower number of visualizations is sufficient to fully represent dynamics since some blocks of the density matrix remain unpopulated during the experiment.

Since we consider Hermitian operators that are invariant under rotations about $\hat{z}$ (termed hereafter zero-quantum operators and denoted ${\hat{\mathrm{ZQ}}}_{\varphi ,m}^{\left(F,K\right)}$ ), a generalized measurement operator in a laboratory frame is written as

4 $$\begin{array}{rl} {\hat{O}}_{\hat{z}} & ={\hat{\mathrm{ZQ}}}_{\varphi ,m}^{\left(F,K\right)}\\ & =\frac{1}{\sqrt{2}}\left(e^{i\varphi }|F,m\rangle \langle K,m|+e^{-i\varphi }|K,m\rangle \langle F,m|\right), \end{array}$$

where the operators are defined up to a phase φ and a projection quantum number m such that |m|≤min⁡(F,K) .

When it comes to a diagonal block (F,F) , one can choose a measurement operator ${\hat{\mathrm{ZQ}}}_{\pi /4,F}^{\left(F,F\right)}$ , which is the same as the maximum-projection operator |F,F〉〈F,F| . The function of the surface representing the (F,F) block can thus be expressed using Eq. ([Disp-formula Ch1.E1]):

5 $${}_{\pi /4,F}^{\left(F,F\right)}r_{\hat{n}}=\mathrm{Tr}\left[\hat{\rho }\;{\hat{R}}_{\hat{z}\to \hat{n}}\;|F,F\rangle \langle F,F|\;{\hat{R}}_{\hat{z}\to \hat{n}}^{-1}\right].$$

One may see that up to this point the presented visualization method is equivalent to the previously described AMPS approach [Bibr bib1.bibx29]; ${}_{\pi /4,F}^{\left(F,F\right)}r_{\hat{n}}$ presented in such a way is also known in the literature as the Husimi Q function (see Appendix G). Note that the visualization is complete in a sense that it fully represents a density matrix for block (F,F) (see Appendix D).

For the representation of a pair of off-diagonal blocks (F,K) and (K,F) , in Eq. ([Disp-formula Ch1.E4]) we choose two measurement operators ${\hat{\mathrm{ZQ}}}_{\varphi ,m}^{\left(F,K\right)}$ such that φ=0 and φ=π/2 (with m chosen from the allowed range). Indeed, given a fixed m in Eq. ([Disp-formula Ch1.E4]), one may notice that the defined operators vary only by one number, φ , thus forming a 2D real operator space. Therefore, it is enough to consider only two values of φ to fully represent the spin dynamics of the coherences in blocks (F,K) and (K,F) .

One can write the function of the surfaces according to Eq. ([Disp-formula Ch1.E1]):

6 $${}_{\;\;\;\varphi ,m}^{\left(F,K\right)}r_{\hat{n}}=\mathrm{Tr}\left[\hat{\rho }\;{\hat{R}}_{\hat{z}\to \hat{n}}\;\;{\hat{\mathrm{ZQ}}}_{\varphi ,m}^{\left(F,K\right)}\;{\hat{R}}_{\hat{z}\to \hat{n}}^{-1}\right],$$

with φ being equal to 0 or π/2 . Similarly to AMPS, the two off-diagonal blocks (F,K) and (K,F) can be fully represented by these two surfaces (see Appendix D). To summarize, the proposed visualization approach is complete in a sense that there is a one-to-one correspondence between the collection of surfaces and blocks of the density operator.

As an example, consider a pair of spin-1/2 nuclei. The density matrix written in the total angular momentum basis consists of four blocks: (1,1) , (1,0) , (0,1) , and (0,0) . For the two diagonal blocks (1,1) and (0,0) , one can consider measurements $(|1,1\rangle \langle 1,1|{)}_{\hat{n}}$ and $(|0,0\rangle \langle 0,0|{)}_{\hat{n}}$ . The two remaining off-diagonal blocks can be represented using the operators ${\hat{\mathrm{ZQ}}}_{0,0}^{\left(1,0\right)}$ and ${\left({\hat{\mathrm{ZQ}}}_{\frac{\pi }{2},0}^{\left(1,0\right)}\right)}_{\hat{n}}$ . Only m=0 is permissible due to the requirement |m|≤min⁡(1,0) . Thereby, one can construct the first measurement operator ${\hat{\mathrm{ZQ}}}_{0,0}^{\left(1,0\right)}=\frac{1}{\sqrt{2}}({\hat{I}}_{1z}-{\hat{I}}_{2z})$ (i.e., the magnetization difference or the so-called zero-quantum in-phase coherence) when φ equals zero and the second operator ${\hat{\mathrm{ZQ}}}_{\frac{\pi }{2},0}^{\left(1,0\right)}=\sqrt{2}\;({\hat{I}}_{1y}{\hat{I}}_{2x}-{\hat{I}}_{1x}{\hat{I}}_{2y})$ when φ equals π/2 (the so-called zero-quantum coherence out of phase; [Bibr bib1.bibx28]).

Examples of different visualized surfaces are presented in Fig. [Fig Ch1.F2]. The first example shown in Fig. [Fig Ch1.F2]a is a polarized state that exemplifies magnetic orientation, i.e., a preferred direction of magnetization in space (see Appendix F for a step-by-step plotting tutorial). The second example in Fig. [Fig Ch1.F2]b shows an anti-phase spin order exemplifying alignment [Bibr bib1.bibx5]. Such a spin order is often an initial state in parahydrogen-based hyperpolarization experiments at a high field [Bibr bib1.bibx11]. These density operators only have nonzero entries within the (1,1) and (0,0) blocks; therefore, the surfaces based on $(|1,1\rangle \langle 1,1|{)}_{\hat{n}}$ measurements fully represent the related density operators (trivially, the (0,0) block is represented by a sphere with a radius 1/4 and therefore is not shown). In Fig. [Fig Ch1.F2]c–d, we give an example of a density operator $\hat{\rho }\;=\;(1/4)\text{𝟙}\;+\;(1/4)({\hat{I}}_{1z}\;-\;{\hat{I}}_{2z})$ that is often encountered in various experiments involving two-spin systems (see the discussion below). Figure [Fig Ch1.F2]c shows the visualized surface based on the measurement $(|1,1\rangle \langle 1,1|{)}_{\hat{n}}$ , which appears isotropic. However, the state is intrinsically anisotropic along the $\hat{z}$ axis, which can be displayed by using a measurement operator ${\left({\hat{\mathrm{ZQ}}}_{0,0}^{\left(1,0\right)}\right)}_{\hat{n}}$ (Fig. [Fig Ch1.F2]d). Through these examples, the usefulness of our visualization approach becomes apparent: one can spot the presence of different symmetries (or lack of thereof) by simply looking at the calculated surface without analyzing the explicit structure of the density matrices.

**Figure 2 Ch1.F2:**
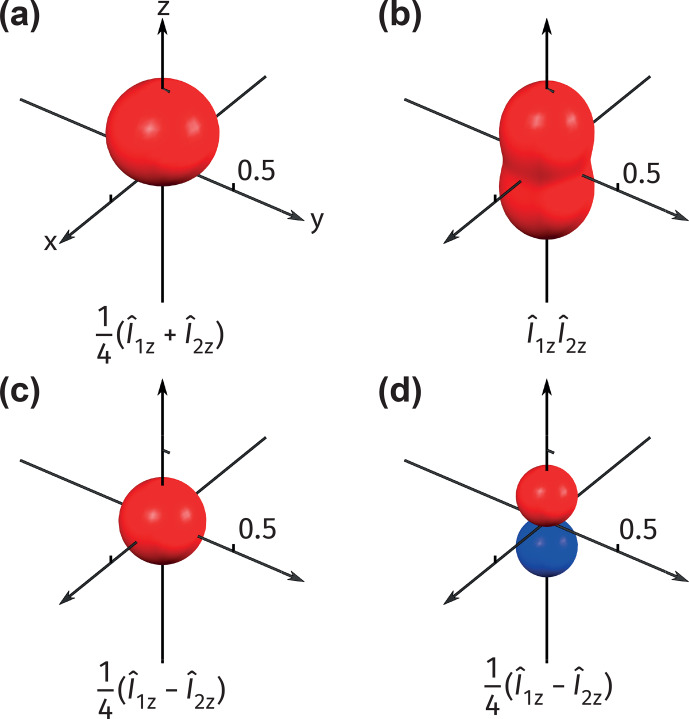
**(a–c)** Visualizations using the measurement observable $(|1,1\rangle \langle 1,1|{)}_{\hat{n}}$ for the following density operators: **(a)** $\hat{\rho }\;=\;(1/4)\text{𝟙}\;+\;(1/4)({\hat{I}}_{1z}\;+\;{\hat{I}}_{2z})$ , representing a state orientated along $\hat{z}$ ; **(b)** $\hat{\rho }\;=\;(1/4)\text{𝟙}\;+\;{\hat{I}}_{1z}{\hat{I}}_{2z}$ , representing a state aligned along $\hat{z}$ ; **(c)** $\hat{\rho }\;=\;(1/4)\text{𝟙}\;+\;(1/4)({\hat{I}}_{1z}\;-\;{\hat{I}}_{2z})$ , representing a state that appears isotropic from the point of measurement of the maximal projection of the total angular momentum with F=1 . This state is intrinsically anisotropic, which is shown in **(d)** by plotting the surface with the measurement operator ${\left({\hat{\mathrm{ZQ}}}_{0,0}^{\left(1,0\right)}\right)}_{\hat{n}}$ .

## 3 Discussion

### 3.1 Equivalence and symmetries

As discussed before, one can easily spot the symmetries of the density operator by looking at corresponding visualized surfaces (Fig. [Fig Ch1.F2]). Furthermore, as shown in Appendix D, the functions $r_{\hat{n}}$ corresponding to surfaces constructed via Eqs. ([Disp-formula Ch1.E5]) and ([Disp-formula Ch1.E6]) contain as much information as the density matrix.

**Figure 3 Ch1.F3:**
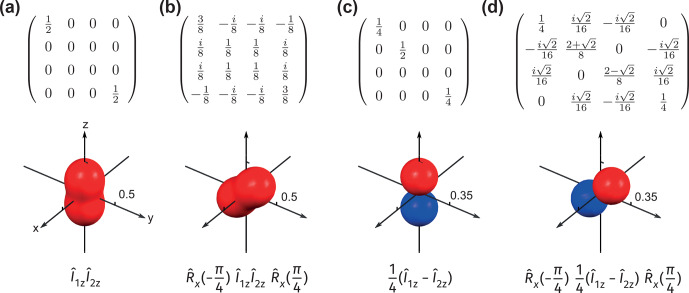
The density operators written in Zeeman basis {|αα〉 , |αβ〉 , |βα〉 and |ββ〉} (top) and their visualizations (bottom) for **(a)** $\hat{\rho }\;=\;\frac{1}{4}\text{𝟙}\;+\;{\hat{I}}_{1z}{\hat{I}}_{2z}$ , **(b)** the same density operator as in panel **(a)** rotated by $-\frac{\pi }{4}$ around $\hat{x}$ , and **(c)** $\hat{\rho }\;=\;\frac{1}{4}\text{𝟙}\;+\;\frac{1}{4}({\hat{I}}_{1z}\;-\;{\hat{I}}_{2z})$ , **(d)** the same density operator as in panel **(c)** rotated by $-\frac{\pi }{4}$ around $\hat{x}$ . Note that the surfaces in panels **(a)**–**(b)** and panels **(c)**–**(d)** are plotted using the measurement operators $(|1,1\rangle \langle 1,1|{)}_{\hat{n}}$ and ${\left({\hat{\mathrm{ZQ}}}_{0,0}^{\left(1,0\right)}\right)}_{\hat{n}}$ , respectively. The rotation is apparent by looking at the plotted surface, while it is hard to understand directly from the matrix form of the density operator.

Now we show explicitly that any global rotation applied to nuclear spins is directly reflected by the rotations of the plotted surfaces. Examples are given in Fig. [Fig Ch1.F3]. A state with alignment (see Fig. [Fig Ch1.F2]b), $\hat{\rho }\;=\;(1/4)\text{𝟙}\;+\;{\hat{I}}_{1z}{\hat{I}}_{2z}$ , is shown in Fig. [Fig Ch1.F3]a; the density operator is fully represented by the surface when plotted using the measurement observable $(|1,1\rangle \langle 1,1|{)}_{\hat{n}}$ . Figure [Fig Ch1.F3]b shows the same density rotated by -(π/4) along $\hat{x}$ . As expected, the visualized surface clearly represents a rotated aligned state. The same logic also applies to the state $\hat{\rho }\;=\;(1/4)\text{𝟙}\;+\;(1/4)({\hat{I}}_{1z}\;-\;{\hat{I}}_{2z})$ (Fig. [Fig Ch1.F3]c–d), for which the visualized surface plotted with $(|1,1\rangle \langle 1,1|{)}_{\hat{n}}$ appears isotropic. Since the state is clearly anisotropic, another measurement operator should be chosen for visualization. A proper choice is ${\left({\hat{\mathrm{ZQ}}}_{0,0}^{\left(1,0\right)}\right)}_{\hat{n}}$ ; the symmetry of the surface is clearly seen when rotation is applied to the density operator (Fig. [Fig Ch1.F3]c–d).

**Figure 4 Ch1.F4:**
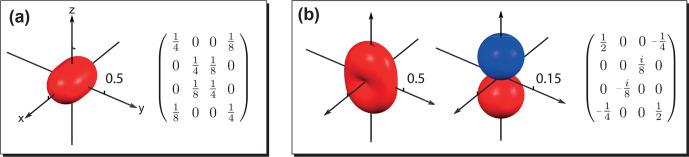
Examples illustrating the relationship between the symmetry of the visualized surface and the elements of the density operator. **(a)** Density operator written in Zeeman basis plotted with $(|1,1\rangle \langle 1,1|{)}_{\hat{n}}$ ; **(b)** density operator written in Zeeman basis plotted with (left) $(|1,1\rangle \langle 1,1|{)}_{\hat{n}}$ and (right) ${\left({\hat{\mathrm{ZQ}}}_{\frac{\pi }{2},0}^{\left(1,0\right)}\right)}_{\hat{n}}$ . In panel **(a)** the surface is 2-fold symmetric around $\hat{z}$ , and the corresponding density operator only has coherences with |Δm|=0 and |Δm|=2 . In panel **(b)**, from left to right, the two surfaces are 2-fold symmetric and rotational symmetric ( q -fold symmetric for an arbitrary positive integer q ) around $\hat{z}$ , and the related density operator (written in Zeeman basis) only has coherences with |Δm|=0 and |Δm|=2 .

In addition, the symmetry of the constructed surfaces can reflect useful information about elements of the density matrix. If a q -fold symmetry is present for all the surfaces, the density matrix, when written with the quantization axis set parallel to the symmetry axis, only has nonzero elements with Δm=qN , where N is an integer (see Appendix E). Examples are given in Fig. [Fig Ch1.F4]. In Fig. [Fig Ch1.F4]a, the only surface-plotted $(|1,1\rangle \langle 1,1|{)}_{\hat{n}}$ representing the state is 2-fold symmetric along $\hat{z}$ . The related density matrix (written in Zeeman basis) only has nonzero coherences with |Δm|=0 and |Δm|=2 . Figure [Fig Ch1.F4]b shows an example where two surfaces are needed to completely represent the state. The one plotted with the measurement operator $(|1,1\rangle \langle 1,1|{)}_{\hat{n}}$ (left) is 2-fold symmetric around $\hat{z}$ , while that plotted with ${\left({\hat{\mathrm{ZQ}}}_{\frac{\pi }{2},0}^{\left(1,0\right)}\right)}_{\hat{n}}$ (middle) is rotationally symmetric ( q -fold symmetric for an arbitrary positive integer q ) around $\hat{z}$ . As a result, both surfaces possess a 2-fold symmetry around $\hat{z}$ , and the related density matrix only has nonzero coherences with |Δm|=0 and |Δm|=2 .

### 3.2 Zero- to ultralow-field nuclear magnetic resonance

First, we show how one could use the presented visualization approach to better understand spin dynamics in ZULF NMR experiments [Bibr bib1.bibx21]. As an example, consider the ${}^{13}$ C-labeled formic acid with ${}^{1}$ H and ${}^{13}$ C spins initially polarized along the z axis (direction of a magnetometer's sensitive axis). During the acquisition a weak bias magnetic field, $B_{x}$ (perpendicular to the magnetometer's sensitive axis), is applied (Fig. [Fig Ch1.F5]a). In such an experiment, nuclear spins evolve due to the heteronuclear J coupling and the bias field, and the ZULF NMR spectrum is collected by measuring the magnetization along the z direction. A simulated NMR spectrum following the described procedure is shown in Fig. [Fig Ch1.F5]b. First, a low-frequency peak is positioned at the average Larmor frequency between the proton and the carbon, $\bar{\nu }=(\nu_{{}^{1}H}+\nu_{{}^{13}C})/2$ ( $\nu_{{}^{1}H}$ and $\nu_{{}^{13}C}$ are the ${}^{1}H$ and ${}^{13}C$ nuclear Larmor frequencies, respectively). In addition, a doublet with splitting equal to the sum of the Larmor frequencies of proton and carbon is centered at the J -coupling frequency.

**Figure 5 Ch1.F5:**
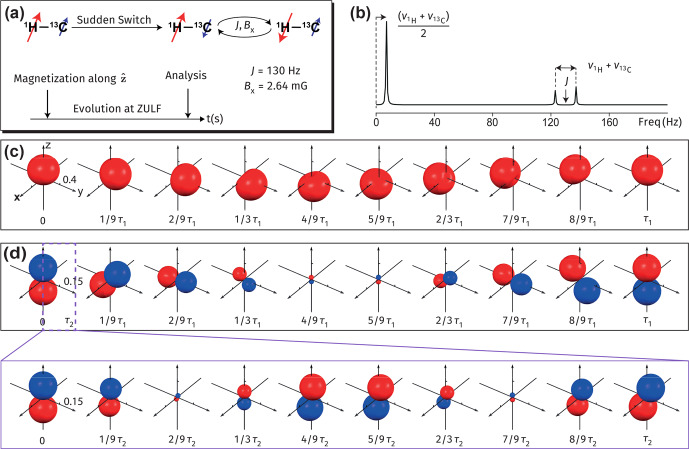
Visualizations of spin dynamics in an AX system ( ${}^{1}$ H– ${}^{13}$ C nuclear pair) during the zero- to ultralow-field nuclear magnetic resonance (ZULF NMR) experiment. **(a)** Scheme of an exemplary ZULF NMR experiment in which a perpendicular field of 2.64 mG is applied during the detection. **(b)** Simulation of the corresponding ZULF NMR spectrum. Assume the initial density operator of the system is ${\hat{\rho }}_{0}\;=\;(1/4)\text{𝟙}\;+\;(1/10)({\hat{I}}_{1z}+4{\hat{I}}_{2z})$ ( ${\hat{I}}_{1}$ and ${\hat{I}}_{2}$ denote ${}^{13}C$ and ${}^{1}H$ nuclei, respectively). Panels **(c)**–**(d)** show surfaces representing spin evolution in the ZULF experiment plotted with the measurements **(c)** $(|1,1\rangle \langle 1,1|{)}_{\hat{n}}$ over a timescale $\tau_{1}=1/\bar{\nu }=2/(\nu_{{}^{1}H}+\nu_{{}^{13}C})$ . Panel **(d)** ${\left({\hat{\mathrm{ZQ}}}_{0,0}^{\left(1,0\right)}\right)}_{\hat{n}}$ over a timescale $\tau_{1}$ (inset) shows the evolution of the surfaces plotted in panel **(d)** over a shorter timescale $\tau_{2}=1/J$ .

The observable operator describing a ZULF measurement is proportional to $\gamma_{{}^{13}C}{\hat{I}}_{1z}+\gamma_{{}^{1}H}{\hat{I}}_{2z}$ (the operators ${\hat{I}}_{1}$ and ${\hat{I}}_{2}$ denote the ${}^{13}C$ and ${}^{1}H$ spins, respectively, and their projections are accordingly ${\hat{I}}_{1z}$ and ${\hat{I}}_{2z}$ ). Further, one can decompose the observable operator into a symmetric part $\left((\gamma_{{}^{13}C}+\gamma_{{}^{1}H})/2\right)({\hat{I}}_{1z}+{\hat{I}}_{2z})$ and an antisymmetric part $\left((\gamma_{{}^{13}C}-\gamma_{{}^{1}H})/2\right)({\hat{I}}_{1z}-{\hat{I}}_{2z})$ . One may notice that symmetric and antisymmetric parts are proportional to the rank-1 component of the measurement operator |1,1〉〈1,1| and the measurement operator ${\hat{\mathrm{ZQ}}}_{0,0}^{\left(1,0\right)}$ , respectively. As a result, the ZULF spectra could be understood by checking the evolution of the surfaces plotted with $(|1,1\rangle \langle 1,1|{)}_{\hat{n}}$ and ${\left({\hat{\mathrm{ZQ}}}_{0,0}^{\left(1,0\right)}\right)}_{\hat{n}}$ measurement operators separately as is done below.

Let us first examine the symmetric part of the measurement operator in the ZULF NMR experiment. Figure [Fig Ch1.F5]c visualizes the evolution of the density operator ${\hat{\rho }}_{0}\;=\;(1/4)\text{𝟙}\;+\;(1/10)({\hat{I}}_{1z}+4{\hat{I}}_{2z})$ (we assume high polarizations and set them proportional to gyromagnetic ratios using $\gamma_{{}^{1}H}/\gamma_{{}^{13}C}\approx 4$ ) by plotting the surface with the operator $(|1,1\rangle \langle 1,1|{)}_{\hat{n}}$ . The resulting oriented surface precesses about the x axis with a period $\tau_{1}=1/\bar{\nu }$ . This motion corresponds to the leftmost peak in Fig. [Fig Ch1.F5]b.

Now let us consider the action of an antisymmetric part of the measurement operator. The intersection with the z axis of the surface plotted in such a way directly corresponds to the observed J doublet (Fig. [Fig Ch1.F5]b). The surface precesses about the x axis with a frequency $\bar{\nu }$ , and its size oscillates with a frequency J (check the corresponding movies to assess the actual evolution of the surface). Such a rotating surface corresponds to a doublet in the ZULF NMR spectrum split around the J frequency (Fig. [Fig Ch1.F5]b).

**Figure 6 Ch1.F6:**
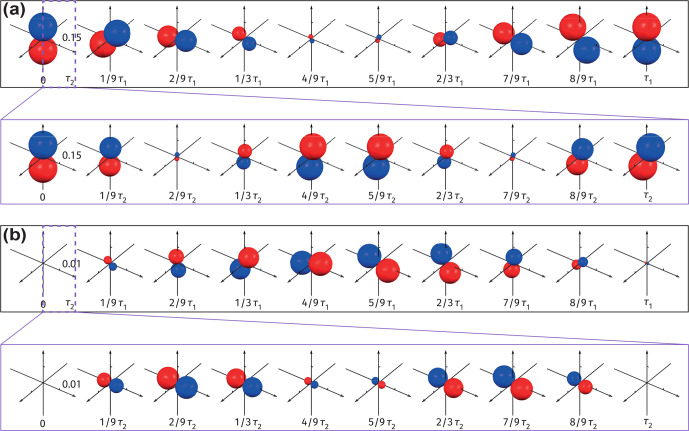
Visualizations of spin dynamics in an AX system ( ${}^{1}$ H– ${}^{13}$ C nuclear pair) during the ZULF experiment. Conditions used for simulation are the same as that used in Fig. [Fig Ch1.F5] except for assuming an initial density operator of **(a)** ${\hat{\rho }}_{0}\;=\;(3/20)({\hat{I}}_{2z}-{\hat{I}}_{1z})$ and **(b)** ${\hat{\rho }}_{0}\;=\;\frac{1}{4}\text{𝟙}\;\;+\;\;\frac{1}{4}({\hat{I}}_{2z}+{\hat{I}}_{1z})$ . Surfaces in panels **(a)** and **(b)** are plotted with the measurement operator ${\left({\hat{\mathrm{ZQ}}}_{0,0}^{\left(1,0\right)}\right)}_{\hat{n}}$ over a timescale $\tau_{1}$ . The insets of panels **(a)** and **(b)** show the evolution of the surfaces over a shorter timescale $\tau_{2}$ . Note the different scaling in panels **(a)** and **(b)**.

In order to understand the asymmetry of the doublet (Fig. [Fig Ch1.F5]b), it is worth analyzing separately the contributions of the symmetric and antisymmetric parts of the initial density matrix to the surface plotted in Fig. [Fig Ch1.F5]d. This is now shown in Fig. [Fig Ch1.F6]a and b, which visualize the evolution of the antisymmetric, ${\hat{\rho }}_{0}\;=\;(3/20)({\hat{I}}_{2z}-{\hat{I}}_{1z})$ , and symmetric, ${\hat{\rho }}_{0}\;=(1/4)\text{𝟙}\;+\;(1/4)({\hat{I}}_{1z}+{\hat{I}}_{2z})$ , parts of the initial density operator, respectively. One can see that the evolution of the asymmetric part of the density operator predominantly contributes to the observed ZULF J spectrum (i.e., Fig. [Fig Ch1.F6]a is almost equivalent to Fig. [Fig Ch1.F5]d); i.e., it gives rise to a symmetric doublet centered at J .

To evaluate this in a more quantitative manner, one can check the intersection of the surface (Fig. [Fig Ch1.F6]a) with the z axis. Given the fact that surfaces plotted with the measurement operator ${\left({\hat{\mathrm{ZQ}}}_{0,0}^{\left(1,0\right)}\right)}_{\hat{n}}$ are rank-1 spherical harmonics (Appendix D), their shapes can be quantified as p orbitals. Considering the evolution of the surface – i.e., the size being proportional to cos⁡(2πJt) and the angle between the p orbital and the z axis being equal to $\left(\pi +2\pi \bar{\nu }t\right)$ – the intersection of the surface with the z axis is found to have the following time dependence, $\mathrm{cos}(2\pi Jt)\mathrm{cos}(\pi +2\pi \bar{\nu }t)=-(1/2)\left[\mathrm{cos}\left(2\pi (J+\bar{\nu })t\right)+\mathrm{cos}\left(2\pi (J-\bar{\nu })t\right)\right]$ , which indeed gives a symmetric doublet centered at J . Similarly, one can check the contribution of the symmetric part of the density matrix (Fig. [Fig Ch1.F6]b) to the measured ZULF J spectrum. In contrast to the surface plotted in Fig. [Fig Ch1.F6]a, the surface plotted in Fig. [Fig Ch1.F6]b appears π/2 radian out of phase in the orientation and -π/2 radian out of phase in the size oscillation. This means that the intersection contributing to the measurement has a time dependence $\mathrm{cos}(-\pi /2+2\pi Jt)\mathrm{cos}(3\pi /2+2\pi \bar{\nu }t)=-(1/2)\left[\mathrm{cos}\left(2\pi (J+\bar{\nu })t\right)-\mathrm{cos}\left(2\pi (J-\bar{\nu })t\right)\right]$ . This term accounts for the small asymmetry of the doublet centered at J shown in Fig. [Fig Ch1.F5]b.

Finally, we note that one also needs the surfaces plotted with the measurement operator ${\left({\hat{\mathrm{ZQ}}}_{\pi /2,0}^{\left(1,0\right)}\right)}_{\hat{n}}$ (Fig. H1) to completely represent the spin system. Since they do not contribute to the ZULF signal measured with one detector, they are not considered here.

### 3.3 Signal amplification by reversible exchange

As a second example, we visualize dynamics of the nuclear spin states of dissolved hydrogen during signal amplification by reversible exchange (SABRE) experiments [Bibr bib1.bibx1] at a high magnetic field [Bibr bib1.bibx6]. The interplay between chemical exchange and coherent spin dynamics is known to induce singlet–triplet mixing [Bibr bib1.bibx18]. For the two protons in free hydrogen (i.e., molecular hydrogen gas dissolved in solution), the singlet–triplet mixing is symmetry-forbidden. However, as soon as the hydrogen molecule binds transiently to a SABRE catalyst and the two protons occupy non-equivalent positions such that chemical symmetry is broken, the difference in their Larmor frequencies, $\Delta =(\delta_{1}-\delta_{2})\gamma B_{0}$ , gives rise to singlet–triplet mixing. Such mixing is selective, and only $|S\rangle \to |T_{0}\rangle$ transitions occur, while the other transitions $|S\rangle \to |T_{\pm }\rangle$ are forbidden at a high field. In the following, we also consider relaxation via locally correlated noise fields to account for the equilibration of triplet sublevels [Bibr bib1.bibx17].

**Figure 7 Ch1.F7:**
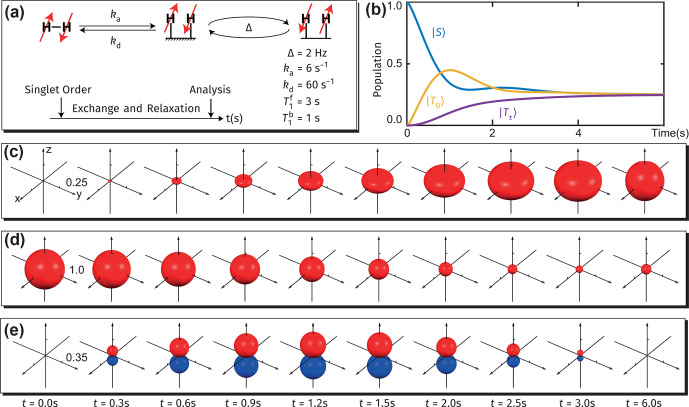
Visualizations of spin dynamics in proton pairs of free molecular hydrogen in a high-field SABRE experiment. **(a)** Scheme of the SABRE experiment: molecular hydrogen initiated in the singlet state undergoes reversible chemical coordination which transiently breaks chemical equivalence between the two spins; NMR, exchange, and relaxation parameters are shown in the inset. **(b)** Evolution of the spin state populations during the SABRE experiment. **(c–f)** Visualization of the evolution under the SABRE experiment plotted with the measurement **(c)** $(|1,1\rangle \langle 1,1|{)}_{\hat{n}}$ , **(d)** the singlet population, and **(e)** ${\left({\hat{\mathrm{ZQ}}}_{\frac{\pi }{2},0}^{\left(1,0\right)}\right)}_{\hat{n}}$ . Note the different scaling in panels **(c)** and **(d)**.

We apply a model recently introduced to simulate spin dynamics in SARBE experiments. All the parameters used for the simulation are given in Fig. [Fig Ch1.F7]a, where the chemical rate constants $k_{a}$ and $k_{d}$ describe the rates of association and dissociation of $H_{2}$ , respectively, and the longitudinal relaxation time of the two protons is given by $T_{1}^{f}$ , when they are free in solution, and $T_{1}^{b}$ , when they are bound to the SABRE catalyst, respectively. The simulated state populations are shown in Fig. [Fig Ch1.F7]b. First, after the start of the exchange process, chemical asymmetry induces the $|S\rangle \to |T_{0}\rangle$ transition and reduces the population difference between the two states. As the $|T_{0}\rangle$ state is populated, relaxation between triplet sublevels becomes more noticeable, and the states $|T_{\pm }\rangle$ also start to be populated. Since the local noise fields are not perfectly correlated, the populations are finally equalized as shown in Fig. [Fig Ch1.F7]b.

The evolving distribution of populations over various states is seen in Fig. [Fig Ch1.F7]c–d. In Fig. [Fig Ch1.F7]c, the AMPS for the triplet block of the density matrix first grow into an oblate spheroid, which indicates that initially only the $|T_{0}\rangle$ state gets overpopulated. Later, the AMPS evolve into a sphere, indicating that all three triplet states are equally populated as a result of relaxation. Figure [Fig Ch1.F7]d shows the changing singlet population. Lastly, Fig. [Fig Ch1.F7]e, which measures the out-of-phase coherence ${\left({\hat{\mathrm{ZQ}}}_{\frac{\pi }{2},0}^{\left(1,0\right)}\right)}_{\hat{n}}$ , reveals extra information not covered by Fig. [Fig Ch1.F7]b; i.e., the singlet–triplet coherence, ${\hat{I}}_{1y}{\hat{I}}_{2x}-{\hat{I}}_{1x}{\hat{I}}_{2y}$ , is transiently formed during the experiment. Since the J coupling between the two protons is ignored, there is no in-phase coherence (i.e., magnetization differences between the two protons along any direction), and the related surface plot is therefore not included here.

### 3.4 Spin-lock-induced crossing

Figure [Fig Ch1.F8] shows a typical spin-lock-induced crossing (SLIC) experiment where a continuous radio frequency (RF) field is applied to a pair of chemically inequivalent nuclear spins at a high field, initially prepared in the singlet spin state. The target spins undergo singlet–triplet conversion if the spins are strongly coupled to each other and the amplitude of the RF field matches the J -coupling value between them [Bibr bib1.bibx13]. The parameters used for the simulation and visualization are shown in Fig. [Fig Ch1.F8]a, where the molecule is specified by the J -coupling strength between the two spins and Δ , the difference between their Larmor frequencies, expressed in hertz. Under the above conditions, the singlet and triplet populations interconvert with a period of $T=\sqrt{2}/\Delta$ .

**Figure 8 Ch1.F8:**
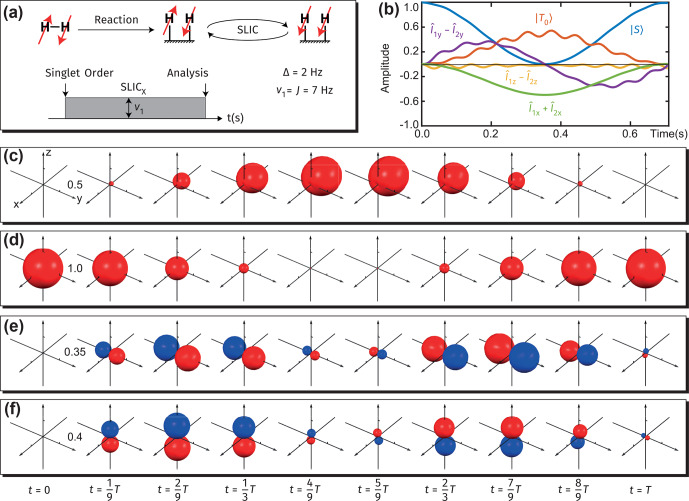
Visualization of spin dynamics in proton pairs during high-field SLIC experiments over a period $T=\sqrt{2}/\Delta$ . **(a)** Scheme of the SLIC experiment: an RF field of the amplitude $B_{1}$ equal to J coupling between the spins is applied along the rotating-frame x axis at the average proton resonance frequency. **(b)** Evolution of various spin orders during the SLIC experiment. **(c–f)** Visualization of the evolution under the SLIC experiment plotted with the measurement **(c)** $(|1,1\rangle \langle 1,1|{)}_{\hat{n}}$ ; **(d)** the singlet population; **(e)** ${\left({\hat{\mathrm{ZQ}}}_{0,0}^{\left(1,0\right)}\right)}_{\hat{n}}$ ; **(f)** ${\left({\hat{\mathrm{ZQ}}}_{\frac{\pi }{2},0}^{\left(1,0\right)}\right)}_{\hat{n}}$ . Note the different scaling in panels **(c)**–**(f)**.

We simulate the SLIC experiment by considering only the coherent processes (no relaxation is included). One could process the simulation results by plotting the population of several chosen operators as a function of time, and the derived population plot is shown in Fig. [Fig Ch1.F8]b (note that all operators are defined in the frame rotating in sync with the RF field). Alternatively, one could observe the dynamics and symmetry of the surfaces visualized via our approach to assess the formation of magnetization and/or alignment states. Figure [Fig Ch1.F8]c illustrates that the singlet spin order is converted – upon application of a SLIC pulse – into the magnetization opposite to the direction of the RF-field amplitude in the rotating frame. Note here that the conversion of the singlet spin order into magnetization parallel to the direction of the RF field is symmetry-allowed but energy-forbidden, which introduces small but fast oscillations observable through the visualized surfaces (Fig. [Fig Ch1.F8]c–f and the movies). Note the formation of in-phase and out-of-phase coherences during application of the SLIC pulse.

### 3.5 Comparison of the generalized measurement-based visualization and DROPS approach

One should note that the earlier DROPS visualization approach [Bibr bib1.bibx15] may also be applied to represent the density operator of strongly coupled nuclear spin systems. For DROPS-based visualization, similar to the present approach, one needs to first decompose the density operator into blocks according to the total angular momentum quantum number F . Then each block (F,K) of the density matrix is expanded in terms of the tensor operator basis (see Eq. B1, according to Eq. C1). Next, one maps the expansion of the density operator into a surface expanded over spherical harmonics such that the function of the surface representing the block (F,K) is

7 $${}^{\left(F,K\right)}r(\theta ,\phi )=\sum_{\lambda ,\mu }\rho_{\lambda \mu }^{\left(F,K\right)}Y_{\lambda \mu }(\theta ,\phi ),$$

where $\rho_{\lambda \mu }^{\left(F,K\right)}$ is the polarization moment and $Y_{\lambda \mu }(\theta ,\phi )$ is the spherical harmonic. The defined radius can be complex. The amplitudes of ${}^{\left(F,K\right)}r(\theta ,\phi )$ are mapped on the radius into the distance of the surface point along the direction specified by (θ,ϕ) , and the phase is mapped into the color of the point according to the color wheel shown in Fig. [Fig Ch1.F9]b.

**Figure 9 Ch1.F9:**
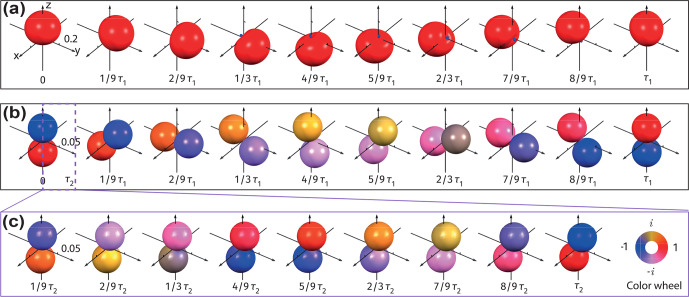
Visualizations of spin dynamics in an AX system ( ${}^{1}$ H– ${}^{13}$ C nuclear pair) during the ZULF experiment using the multipole tensor operator-based DROPS approach. Conditions are the same as those used in Fig. [Fig Ch1.F5]. Panels **(a)**–**(c)** show the surfaces representing the spin evolution during the ZULF experiment of **(a)** the (1,1) block and **(b)** the (1,0) block. The inset shows the evolution of the surface plotted in panel **(b)** over a shorter timescale of $\tau_{2}$ . Colors of surfaces denote the phase of the calculated radius (Eq. [Disp-formula Ch1.E7]) according to the color wheel shown at the bottom right.

As discussed in the Results section, for a system of N -coupled spins-1/2, the density matrix written in a total angular momentum basis should have in total $h^{2}$ blocks: $h=C_{N}^{N/2}$ (binomial coefficient) when N is even and $h=C_{N}^{(N-1)/2}$ when N is odd. Given the hermiticity of the density operator, only one of blocks (F,K) or (K,F) needs to be visualized in the DROPS approach. Therefore, only h(h+1)/2 surfaces are needed to fully represent the density operator.

To compare the DROPS approach with the generalized measurement-based visualization approach, we look at the spin dynamics in an AX system during the ZULF experiment once again (Fig. [Fig Ch1.F9]). Figure [Fig Ch1.F9]a shows the evolution of the surface representing the (1,1) block, which precesses about the x axis with the frequency $\bar{\nu }$ . It resembles the AMPS shown in Fig. [Fig Ch1.F5]c apart from a small “blue tail” observed here. Modeling shows this tail to be related to the rank-2 component of the density operator. The (1,0) block is represented through the surface shown in Fig. [Fig Ch1.F9]b. The color of the surface changes periodically with the frequency J , and the orientation precesses about the x axis with the frequency $\bar{\nu }$ . While the surface also captures the full dynamics and directly reflects the two characteristic frequencies, J and $\bar{\nu }$ , of the system, it is less obvious how to quantify the measured ZULF J spectra from it.

## 4 Conclusions

In this paper, we extend an angular momentum probability surfaces (AMPS) approach for visualizing dynamics of quantum spin systems based on generalized observable operators. The plotted 3D surfaces conveniently represent symmetries of density matrices and allow spotting of their presence (orientation, alignment, etc.) or absence even when direct analysis of (time-dependent) density-matrix elements is not obvious. Three different experiments are used to demonstrate the applicability of the novel visualization approach: (i) evolution of the heteronuclear ${}^{1}$ H– ${}^{13}$ C spin pair during the zero- to ultralow-field (ZULF) NMR experiment; (ii) physicochemical conversion of parahydrogen during the signal amplification by reversible exchange (SABRE) experiment at a high magnetic field; (iii) spin dynamics in the ensembles of pairs of spin-1/2 nuclei during the spin-lock-induced crossing (SLIC) experiment. The presented approach allows visualization of complex dynamics in multi-spin systems and may find applications for describing hyperpolarization experiments utilizing parahydrogen (PHIP and SABRE) and general NMR experiments, especially under zero- to ultralow-field conditions.

## Supplement

10.5194/mr-3-145-2022-supplement The supplement related to this article is available online at: https://doi.org/10.5194/mr-3-145-2022-supplement.

## Data Availability

No data sets were used in this article.

## Code availability

The software code for the graphics shown in this paper is available in the Supplement. For exporting figures from the Matlab simulation software, the export_fig (https://github.com/altmany/export_fig/releases/tag/v3.27, last access: 15 May 2022, Altman, 2022) toolbox was used.

## Video supplement

Video representations of the figures are available in the Supplement.

## Appendix

The supplement related to this article is available online at: https://doi.org/10.5194/mr-3-145-2022-supplement.
