# Supplementary material for: Visualization of dynamics in coupled multi-spin systems
Source: Magn Reson (Gott). 2022 Aug 9;3(2):145–60. doi: 10.5194/mr-3-145-2022 (PMC10583293; doi:10.5194/mr-3-145-2022)
Supplement: The supplement related to this article is available online at: https://doi.org/10.5194/mr-3-145-2022-supplement. [file mr-3-145-supplement.zip › mr-3-145-2022-supplement-title-page.pdf]

Supplement of Magn. Reson., 3, 145–160, 2022
https://doi.org/10.5194/mr-3-145-2022-supplement
© Author(s) 2022. CC BY 4.0 License.

*Supplement of*

# Visualization of dynamics in coupled multi-spin systems

**Jingyan Xu et al.**

*Correspondence to:* Danila A. Barskiy (dbarskiy@uni-mainz.de)

- mr-3-145-2022-supplement-title-page.pdf
- code.zip
- video.zip

The copyright of individual parts of the supplement might differ from the article licence.
